# Versatile Fungal Polyphenol Oxidase with Chlorophenol Bioremediation Potential: Characterization and Protein Engineering

**DOI:** 10.1128/AEM.01628-18

**Published:** 2018-11-15

**Authors:** Efstratios Nikolaivits, Maria Dimarogona, Ioanna Karagiannaki, Angelina Chalima, Ayelet Fishman, Evangelos Topakas

**Affiliations:** a Biotechnology Laboratory, School of Chemical Engineering, National Technical University of Athens, Athens, Greece; b Department of Chemical Engineering, University of Patras, Patras, Greece; c Department of Biotechnology and Food Engineering, Technion-Israel Institute of Technology, Haifa, Israel; d Biochemical and Chemical Process Engineering, Division of Sustainable Process Engineering, Department of Civil, Environmental and Natural Resources Engineering, Luleå University of Technology, Luleå, Sweden; McMaster University

**Keywords:** Pichia pastoris, Thermothelomyces thermophila, chlorophenol bioremediation, polyphenol oxidase, protein engineering, tyrosinase

## Abstract

Polyphenol oxidases (PPOs) have been mostly associated with the undesirable postharvest browning in fruits and vegetables and have implications in human melanogenesis. Nonetheless, they are considered useful biocatalysts in the food, pharmaceutical, and cosmetic industries. The aim of the present work was to characterize a novel PPO and explore its potential as a bioremediation agent. A gene encoding an extracellular tyrosinase-like enzyme was amplified from the genome of Thermothelomyces thermophila and expressed in Pichia pastoris. The recombinant enzyme (*Tt*PPO) was purified and biochemically characterized. Its production reached 40 mg/liter, and it appeared to be a glycosylated and N-terminally processed protein. *Tt*PPO showed broad substrate specificity, as it could oxidize 28/30 compounds tested, including polyphenols, substituted phenols, catechols, and methoxyphenols. Its optimum temperature was 65°C, with a half-life of 18.3 h at 50°C, while its optimum pH was 7.5. The homology model of *Tt*PPO was constructed, and site-directed mutagenesis was performed in order to increase its activity on mono- and dichlorophenols (di-CPs). The G292N/Y296V variant of *Tt*PPO 5.3-fold increased activity on 3,5-dichlorophenol (3,5-diCP) compared to the wild type.

**IMPORTANCE** A novel fungal PPO was heterologously expressed and biochemically characterized. Construction of single and double mutants led to the generation of variants with altered specificity against CPs. Through this work, knowledge is gained regarding the effect of mutations on the substrate specificity of PPOs. This work also demonstrates that more potent biocatalysts for the bioremediation of harmful CPs can be developed by applying site-directed mutagenesis.

## Abstract (precis)

A novel fungal PPO was heterologously expressed and biochemically characterized. Construction of single and double mutants led to the generation of variants with altered specificity against CPs. Through this work, knowledge is gained regarding the effect of mutations on the substrate specificity of PPOs. This work also demonstrates that more potent biocatalysts for the bioremediation of harmful CPs can be developed by applying site-directed mutagenesis.

## INTRODUCTION

Polyphenol oxidases (PPOs) exhibit two enzymatic activities, tyrosinase (l-tyrosine, l-dopa:oxygen oxidoreductase; EC 1.14.18.1) and catechol oxidase (1,2-benzenediol:oxygen oxidoreductase; EC 1.10.3.1) that belong to the type III copper family, along with hemocyanins, which display no enzymatic activity. The type III copper center consists of two copper ions (CuA and CuB) coordinated by three histidine residues each (1). The distinction between tyrosinases and catechol oxidases was thought to exist in their ability to hydroxylate phenols in the *ortho* position (monophenolase; cresolase activity) and more specifically tyrosine. However, recent advances in the field show that the hydroxylase activity should not be correlated with the commonly used substrates of tyrosinases (tyrosine and tyramine) and that it is a general function of PPOs (2).

PPOs are found in all domains of life, are distributed from bacteria to humans, and their main role is the formation of melanins and other phenolic polymers, mainly for protective purposes (3). In different animal phyla, melanins are formed by various types of precursors and are located in different parts, as follows: mostly (but not exclusively) in the skin, hair, and eyes of mammals; in bird feathers; on skin (but also extracutaneous regions) in reptiles, amphibians, and fish; and in the exoskeleton of insects (4).

In plants and mushrooms (basidiomycetes), PPOs have been well studied, due to the undesirable postharvest browning they cause, which downgrades the value of these products (3, 5). Plant PPOs have also been found to take part in secondary metabolism for the synthesis of polyphenols (betalain, lignans, and aurones) (5). Other functions that have to do with l-tyrosine metabolism (in walnut) can lead to leaf necrosis phenotypes not associated with pathogens (6). On the other hand, PPOs in plants have been associated with herbivore and pathogen resistance (7). Bacterial tyrosinases are either extracellular (e.g., from Streptomyces and Bacillus strains) or intracellular (from Marinomonas mediterranea) and are induced by heat or the presence of l-tyrosine (3, 4).

While the pathway for melanin production in basidiomycetes is more similar to that of animals, melanogenesis in ascomycetes is correlated with specific developmental stages (mycelium, sporulation, and wounding) and is located in the cell wall. In most cases, melanin precursors (hydroxylated naphthalene derivatives, not tyrosine) are secreted by the ascomycete and then oxidized extracellularly (4). Melanin functions as a protection factor against radiation, temperature, reactive oxygen species, pesticides, and pathogens, but it can also act as a virulence factor of microbial cells against their host (4).

Several PPOs have been heterologously expressed in various hosts. For example, human tyrosinase has been expressed in two different types of insect cells, as well as in Escherichia coli (8, 9). Tyrosinase cDNA from the pearl oyster Pinctada fucata was expressed in an E. coli cell-free system (10). Plant PPOs from the flower Coreopsis grandiflora (11) and the grape Vitis vinifera (12) were functionally expressed in E. coli, whereas PPOs from the tea tree Camellia sinensis were poorly expressed in both prokaryotic and eukaryotic hosts (13). When it comes to Basidiomycota, several PPOs from Agaricus bisporus have been expressed in E. coli (14, 15) and Saccharomyces cerevisiae (16), while recently, a tyrosinase from Polyporus arcularius was expressed in E. coli cells (17). Bacterial tyrosinases from various genera (Verrucomicrobium, Bacillus, Rhizobium, Ralstonia, and Marinomonas) have been heterologously expressed and characterized (18–22). Finally, only a few PPOs from Ascomycota have been expressed in heterologous hosts; several from Aspergillus oryzae expressed in Trichoderma reesei (23), Yarrowia lipolytica (24), and E. coli (25) and a Trichoderma reesei tyrosinase were expressed in Pichia pastoris (26).

Mechanistic and structural studies of tyrosinase are important for developing potent inhibitors for use in hyperpigmentation-associated diseases in humans (27). Other than that, PPOs have been used as biocatalysts in many reactions, with various applications in food, pharmaceutical, and cosmetic industries (28, 29) and also as biosensors for the detection of small amounts of phenolics in contaminated waters (30). Furthermore, they have shown potential in the bioremediation of wastewaters containing phenolic contaminants and for the reduction of chemical oxygen demand (28, 31, 32). As recently reviewed, although laccases have been widely studied, the class of tyrosinases remains underinvestigated, especially concerning its application in the bioremediation of phenolics (33).

Chlorophenols (CPs) are common organic pollutants introduced in the environment by the activities of various industries and are mainly associated with the production, use, and degradation of several pesticides. CPs may also be produced when wastewater or drinking water is disinfected with chlorine under certain conditions (34). Some CPs have been listed by the US Environmental Protection Agency as priority contaminants, as they impose many health risks for living organisms, like DNA damage, oxidative stress, toxicity, and carcinogenicity (35). Enzymatic bioremediation of these pollutants is often superior to microbial bioremediation, due to the higher tolerance of enzymes for concentrated CPs.

Myceliophthora thermophila (synonym Sporotrichum thermophile) is a thermophilic filamentous ascomycete fungus that was recently repositioned to the Thermothelomyces genus (36). This genus also includes the industrially important strain C1 (formerly known as Chrysosporium lucknowense C1) (37). T. thermophila is a very strong lignocellulose degrader expressing a wide variety of relevant enzymes. Since the elucidation of its genome sequence (DOE Joint Genome Institute; http://genome.jgi.doe.gov/), many new enzymes implicated in the hydrolysis of cellulose and hemicellulose have been discovered and studied (38). Concerning oxidoreductases, only a few have been studied so far, as follows: some lytic polysaccharide monooxygenases (LPMOs) (39–41), a P450 fatty-acid monooxygenase (42), a xylooligosaccharide oxidase (43), two vanillin alcohol-type oxidases (44), a peroxidase (45), and a well-known commercially available laccase (46).

The present work reports the discovery, cloning, and expression of a tyrosinase-like gene (*Tt60685*) from the genome of T. thermophila in P. pastoris. Some expression conditions, such as induction temperature, medium, and copper concentration, were studied with the aim of achieving higher protein production. The recombinant protein (*Tt*PPO) was purified and biochemically characterized in terms of substrate specificity and effect of temperature and pH on its activity. Three single mutations and their combinations were introduced to the amino acid sequence of *Tt*PPO, and the mutants were characterized and evaluated for their ability to transform various mono- and di-CPs. The purpose of this study was dual, to create a more potent biocatalyst for the bioremediation of CPs and to gain better insight into amino acid residues which control the specificity of PPOs.

## RESULTS

### Cloning and sequence analysis of the putative PPO.

The open reading frame (ORF) of the *Tt60685* gene from T. thermophila (GenBank accession no. NC_016477.1) contains an intron of 219 bp and encodes a putative tyrosinase-like enzyme of 424 amino acids, including a signal peptide of 22 amino acids (MKPAALLGAALAAVAFPAGAHA). Alignment of this protein sequence (https://blast.ncbi.nlm.nih.gov/Blast.cgi) with ones from the Protein Data Bank (PDB) revealed the highest similarity (45%) with the catechol oxidase from Aspergillus oryzae (23), followed by bacterial tyrosinases from Streptomyces castaneoglobisporus (47) and Bacillus megaterium (48), with identities of 28% and 26%, respectively. A phylogenetic tree was constructed after aligning these protein sequences using the maximum likelihood method (see Fig. S1 in the supplemental material). The tree is drawn to scale, with branch lengths measured in the number of substitutions per site. The results suggest that *Tt*PPO belongs to the same branch as the catechol oxidase from A. oryzae, with the highest identity (45%). Moreover, these ascomycete PPOs are more similar to bacterial tyrosinases than ascomycete tyrosinases, which are located in the same branch as basidiomycete PPOs. As expected, plant PPOs form a separate family, which is closer to the ascomycete PPOs and bacterial tyrosinases than ascomycete tyrosinases and basidiomycete PPOs.

The cDNA of the putative tyrosinase-like gene was cloned in the P. pastoris expression vector pPICZαA under regulation of the *AOX1* promoter. The mature protein had a predicted molecular weight of 46,464 Da and pI of 5.34, while it showed 3 potential *N*-glycosylation and 7 potential *O*-glycosylation sites.

### Expression and purification of *Tt*PPO.

Zeocin-resistant transformants were plated on a minimal medium methanol-containing agar plate and were grown for 3 days. Subsequently, a plate assay was performed, and 4-chlorocatechol-oxidizing colonies were identified by the dark-brown color appearing on and around the colonies, in contrast to the wild-type strain that showed no oxidizing activity (Fig. S2).

Seven high-color-forming colonies were picked and cultured in 50 ml liquid medium. Culture samples were taken each day, and the extracellular enzymatic activity and cell growth were measured. The enzyme activity reached a peak on the 4th day after induction, while the wild-type X-33 strain showed no activity. The highest-enzyme-producing colony was used for further expression studies.

The availability of copper during the production of the recombinant *Tt*PPO may prove an important factor affecting the activity of the produced protein, as it contains two copper ions per protein molecule. Therefore, the effect of different CuSO_4_ concentrations (0 to 100 μM) added to the culture medium on the enzymatic activity was studied. The highest activity was observed at 25 μM CuSO_4_, while in the absence of added copper ions, the activity was very low. The effect of the culture medium and induction temperature on the activity of the produced recombinant enzyme indicated that a complex methanol-containing buffered medium (BMMY; EasySelect *Pichia* expression kit) and low induction temperature (23°C) resulted in higher PPO activity.

**FIG 1:**
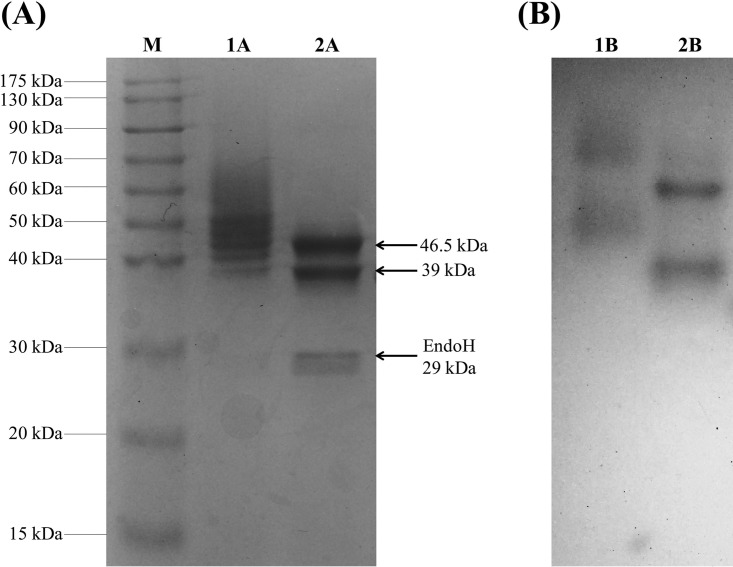
SDS-PAGE gels of the IMAC-purified *Tt*PPO. Samples were run under either denaturing (A) or native (B) conditions. Lane M, prestained protein marker; lanes 1A and 1B, untreated *Tt*PPO; lanes 2A and 2B, endoglycosidase H-treated *Tt*PPO.

**FIG 2:**
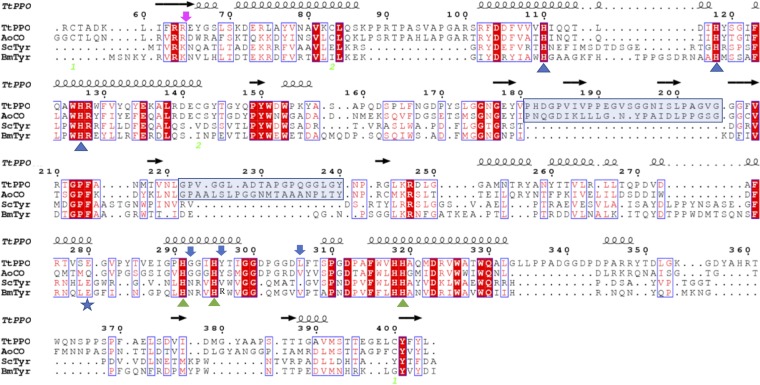
Structure-based sequence alignment of *Tt*PPO, *Ao*CO (PDB ID 4J3R), *Sc*Tyr (PDB ID 1WX2), and *Bm*Tyr (PDB ID 3NM8). Copper-coordinating histidines are indicated by a blue triangle (CuA) and a green triangle (CuB). The residues that have been mutated are denoted by a blue arrow. The potential cleavage site at the N terminus is indicated by a magenta arrow. The blue frames denote the insertions present in *Tt*PPO and *Ao*CO sequences.

The recombinant protein was purified with immobilized metal ion affinity chromatography (IMAC) from the concentrated cell-free culture broth, with a yield of 39.4 mg · liter^−1^ pure protein. The homogeneity of the purified enzyme was checked with SDS-PAGE, where *Tt*PPO appears as a smeared band, with several distinguished bands ranging from 39 to 50 kDa (Fig. 1A). After deglycosylation with endoglycosidase H, which acts on *N*-glycosylation sites, all these bands merge to just two, with one at 46.5 kDa and one at 39 kDa. The upper band corresponds to the theoretical molecular weight of *Tt*PPO. The lower band is approximately 7.5 kDa less than the calculated molecular weight. The truncated amino acids most probably originate from the N terminus of the protein, as the His tag-containing C terminus must be intact, since the protein was retained on the affinity column. The activity staining on a native gel (Fig. 1B) showed that both bands are active prior to (lane 1B) and after (lane 2B) deglycosylation. The truncated form of *Tt*PPO may be the product of the Kex2 endoprotease, which is produced by P. pastoris strain in order to cleave the α-factor signal sequence. This peptidase recognizes the amino acid sequence N–K/R-R–C and cleaves at its carboxyl end. It is assumed that this protease cleaves not only at the signal sequence but also after the 64th amino acid of the mature protein, recognizing a second cleavage site (R-R; magenta arrow in Fig. 2). In that case, the resulting protein lacks 7.2 kDa, which is approximately the difference seen on the SDS gel. Further studies were performed with the mixture of the full and truncated forms of *Tt*PPO, since both proved to be active.

### Characterization of purified *Tt*PPO.

**FIG 3:**
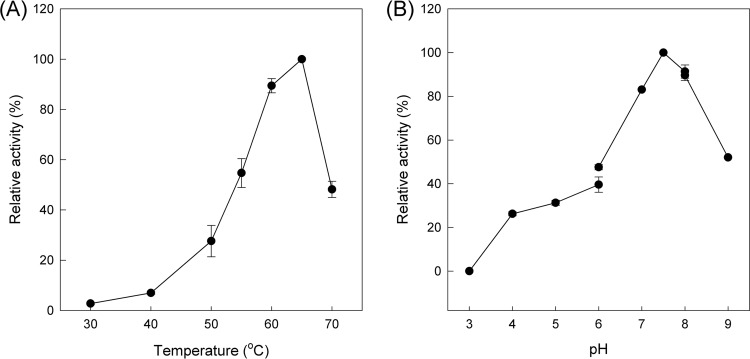
Effect of temperature (A) and pH (B) on the activity of the purified recombinant enzyme. The enzyme concentration used was 8.6 μg · ml^−1^. Error bars represent the standard deviation from independent biological triplicates.

**FIG 4:**
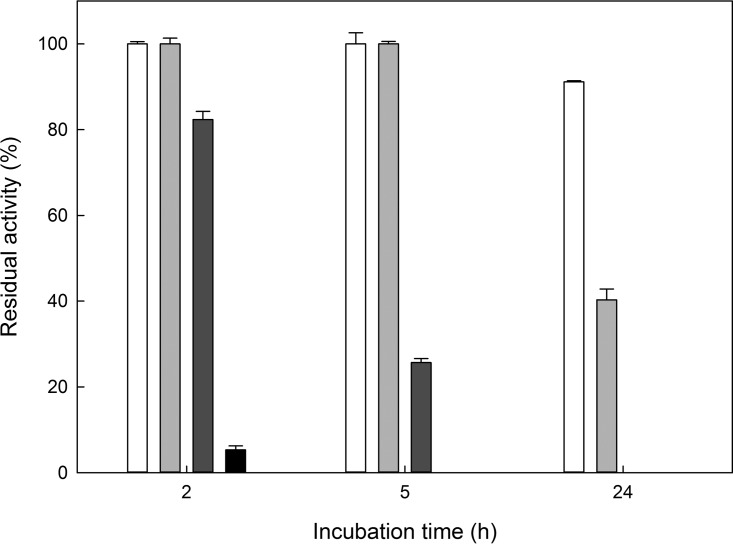
Effect of temperature on the stability of the recombinant enzyme at 40°C (white), 50°C (light gray), 60°C (dark gray), and 70°C (black). The enzyme was incubated at a concentration of 1.1 mg · ml^−1^. Error bars represent the standard deviation from independent biological triplicates.

The temperature at which the recombinant PPO showed its maximum activity was 65°C (Fig. 3A), but it also showed satisfactory activity (over 50%) at temperatures ranging from 55°C to 70°C. Extensive incubation of *Tt*PPO at those temperatures resulted in a loss of over 70% of its initial activity at 60°C and over 90% at 70°C after 5 and 2 h of incubation, respectively (Fig. 4). At lower temperatures, the enzyme was stable for at least 5 h and suffered minor losses (<10%) after 24 h at 40°C, while its half-life was 18.3 h and 2.5 h at 50°C and 60°C, respectively. *Tt*PPO activity was optimal at a range of pH 7 to 8 (maximum, 7.5), while it retained a significant part of its activity (over 50%) at pH values from 6 to 9 (Fig. 3B).

The recombinant *Tt*PPO proved to be a versatile enzyme able to oxidize a wide range of phenolic substrates. Kinetic constants were determined for seven substrates and are presented in Table 1. *Tt*PPO showed the highest affinity for 4-chlorocatechol, followed closely by vanillin and l-3,4-dihydroxyphenylalanine (l-DOPA). The highest *K_m_* values were found for hydroquinone, catechin, and catechol. On the other hand, the enzyme showed very high activity (*k*_cat_) on catechin, at 7 times higher than for 4-chlorocatechol.

**TABLE 1:** Kinetic constants of *Tt*PPO on phenolic substrates^*a*^

Substrate	*k*_cat_ (min^−1^)	*K_m_* (mM)	*k*_cat_/*K_m_* (min^−1^ · mM^−1^)
Catechol	131.4 (6.4)	27.0 (2.8)	4.9 (0.6)
4-Chlorocatechol	341.6 (9.7)	1.6 (0.1)	209.5 (14.8)
l-DOPA	6.5 (0.8)	2.2 (0.6)	3.0 (0.9)
Catechin	2,428.7 (824.0)	31.0 (12.9)	78.3 (42.0)
Vanillin	115.3 (3.4)	2.0 (0.1)	59.2 (4.1)
Guaiacol	174.3 (9.7)	4.2 (0.6)	41.4 (6.1)
Hydroquinone	101.2 (5.9)	54.4 (5.5)	1.9 (0.1)

a Numbers in parentheses are the standard deviation from independent biological duplicates.

Besides these substrates, the recombinant enzyme could also rapidly oxidize six additional compounds, including catecholic, biphenyl, and naphthalene derivatives (Table 2). Furthermore, *Tt*PPO showed low activity on catechol, guaiacol, and phenol derivatives, including cresols (Table 3), which have potential harmful effects on human health. Additionally, the enzyme showed limited activity on one nonphenolic substrate (veratryl alcohol). The structures of all substrates that could be oxidized by *Tt*PPO are summarized in Fig. S3. Overall, *Tt*PPO could oxidize 28 out of the 30 substrates tested.

**TABLE 2:** Substrate specificity of *Tt*PPO on various phenolic substrates

Substrate^*a*^	Specific activity (SD) (U/g)
3,5-Dichlorocatechol	174.4 (8.9)
Caffeic acid	328.0 (7.5)
Epinephrine	128.7 (2.8)
2,3-Dihydroxybiphenyl	192.8 (1.8)
Pyrogallol	672.7 (43.7)
1,8-Dihydroxynaphthalene	621.7 (2.6)

a Concentration of 5 mM under standard assay conditions.

**TABLE 3:** Relative activity of *Tt*PPO on various substrates compared to the activity on catechol^*a*^

Substrate^*b*^	Relative activity (%)
Polyphenols	
Epicatechin	140
Quercetin	59
Catechol derivatives	
Protocatechuic acid	11
Benzene-triol derivatives	
Gallic acid	20
Guaiacol derivatives	
Vanillic acid	32
Ferulic acid	11
Syringol	12
Hydroxyphenyl derivatives	
Resorcinol	1
Gentisic acid	3
Phenol derivatives	
*o*-Cresol	23
*p*-Cresol	11
*p*-Hydroxybenzoic acid	3
*p*-Hydroxyphenylacetic acid	0
*p*-Tyrosol	10
l-Tyrosine	0
d-Tyrosine	1
Nonphenolics	
Veratryl alcohol	14

a Specific activity for catechol is 0.34 U/mg.

b Substrate concentration was 5 mM. The reaction terminated after 20 h at 35°C.

### Construction of homology model and selection of point mutations.

**FIG 5:**
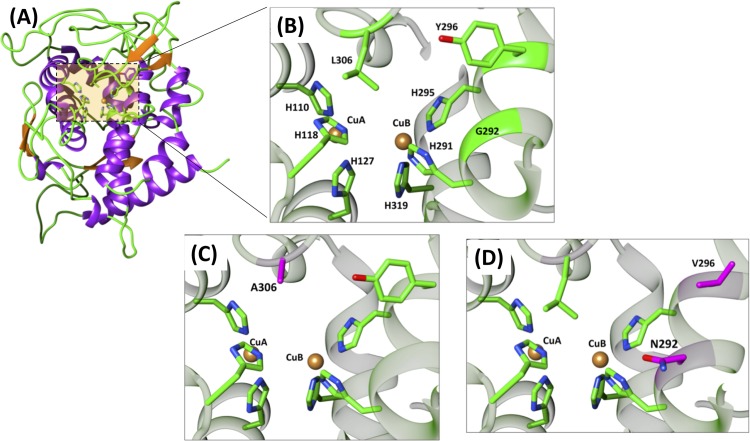
(A) Ribbon representation of overall *Tt*PPO structural model. The active site is shown in the beige square. (B) Enlarged representation of active site. Coordinating histidines and amino acids targeted for mutation are indicated by green sticks. (C) Active site of L306A variant. (D) Active site of G292N/Y296V variant.

The full-length sequence of recombinant *Tt*PPO was submitted to the HHpred server for identification of homologues with known structures. The top scoring PDB entry was the crystal structure of A. oryzae catechol oxidase (*Ao*CO, PDB ID 4J3R). *Ao*CO shares 45% sequence identity for 82% coverage with *Tt*PPO and has an E value practically equal to zero. It was therefore chosen as the template for the construction of a *Tt*PPO structural model by Modeller (Fig. 5). The second closest PPO with a known structure is Streptomyces castaneoglobisporus tyrosinase (*Sc*Tyr, PDB ID 1WX2, 27% identity [ID] for 63% sequence [seq] coverage), followed by Bacillus megaterium tyrosinase (*Bm*Tyr, PDB ID 3NM8, 26% ID for 64% seq coverage).

Similarly to *Ao*CO, *Tt*PPO belongs to the “short” tyrosinase-like enzyme group lacking the C-terminal domain (49). It features the conserved arginine residue (R63) and the tyrosine motif (Y401-F402-Y403), which in the case of plant and fungal PPOs marks the end of the catalytic domain and the beginning of the linker region. These residues are situated in two parallel beta-sheets and bridge the N and C termini of the polypeptide chain by forming pi-cation and beta-sheet interactions (50). The two copper ions are coordinated by 6 histidine residues, as follows: His110 (H_A1_), His118 (H_A2_), and His127 (H_A3_) for CuA, and His295 (H_B1_), His291 (H_B2_), and His319 (H_B3_) for CuB (Fig. 5). Among the three disulfide bridges observed in the *Ao*CO structure, two seem to be conserved in *Tt*PPO between Cys55 and Cys400 and between Cys83 and Cys142. *Tt*PPO, similarly to *Ao*CO, contains two insertions (residues 181 to 205 and 222 to 240) that are missing from the *Sc*Tyr and *Bm*Tyr structures (Fig. 2). These additional amino acids form extended loop regions above the copper-coordinating sites, restricting access to the active site.

With the aim to decipher the structural characteristics that influence PPO functional properties, previous studies have focused on various residues located in the vicinity of the active site (12, 51). Significant variations are observed in the region around CuA which are thought to determine enzyme specificity (1). Accessibility to the CuA site was previously hypothesized to be a determining factor for monophenolase activity (49). In *Tt*PPO, the CuA site is shielded by a leucine residue (L306). To examine the effect of this residue on *Tt*PPO activity, L306 was mutated to alanine (Fig. 5C).

Regarding residues surrounding CuB, R209 in *Bm*Tyr, which is located after H_B2_, was suggested to be critical for substrate orientation in the active site (48). The corresponding residue in *Tt*PPO is a tyrosine (Y296), similarly to *Ao*CO, while in *Sc*Tyr, the corresponding residue is a valine. Another potential determinant of PPO substrate specificity is the residue located next to the CuB-coordinating H_B1_. It has been shown that the occurrence of an asparagine residue in this location may determine monophenolase activity (12). In the case of *Tt*PPO, the corresponding residue is a glycine (G292), similar to *Ao*CO. To study the role of the aforementioned residues in the biochemical function of *Tt*PPO, the Y296V and G292N mutants were expressed and characterized (Fig. 5B and D).

### Oxidation of CPs by variants of *Tt*PPO.

**FIG 6:**
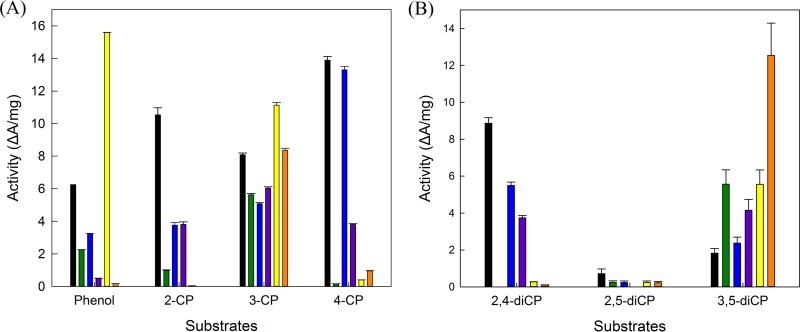
Activity of *Tt*PPO (black; 43 μg · ml^−1^) and its mutants, the G292N variant (green; 77 μg · ml^−1^), L306A variant (blue; 52 μg · ml^−1^), Y296V variant (purple; 52 μg · ml^−1^), G292N/L306A variant (yellow; 50 μg · ml^−1^), and G292N/Y296V variant (orange; 54 μg · ml^−1^) measured after 16 h at 35°C on phenol, mono-CPs (A), and di-CPs (B). Error bars represent the standard deviation from independent biological duplicates.

Based on the colorimetric screening, wild-type *Tt*PPO showed activity on phenol and various mono- and di-CPs, with the highest activity on 4-CP and the lowest on 2,5-dichlorophenol (2,5-diCP). The L306A mutant also exhibited the highest activity on 4-CP, while the other single mutants (G292N and Y296V) were most active on 3-CP (Fig. 6A). Double mutants showed shifted specificity, with the G292N/L306A mutant toward phenol and the G292N/Y296V mutant toward 3,5-diCP. Compared to the wild type, these variants also showed higher activity on 3-CP and 3,5-diCP, respectively.

In order to quantify the depletion of CPs in each reaction, high-performance liquid chromatography (HPLC) analysis was performed. 2,5-diCP was excluded from further studies because of the low reactivity of all *Tt*PPO variants against it. Wild-type *Tt*PPO seemed to be the best candidate for the removal of 2-CP, 4-CP, and 2,4-diCP, with bioconversion yields of 35.4% ± 0.2%, 25.2% ± 0.3%, and 25.5% ± 1.5%, respectively. In the case of 3-CP, even though the preliminary screening showed that the G292N/L306A variant exhibits 1.4-fold-increased activity, quantification of the remaining 3-CP for this variant and the wild-type showed similar yields (21.0% ± 0.0% and 19.7% ± 0.2%, respectively). However, in the case of the wild-type reaction, there seems to be residual chlorocatechol (23.3 ± 0.1 μM), as identified by a standard 4-chlorocatechol solution (Fig. S4A). That may imply that the activity on 4-chlorocatechol of this mutant is increased compared to the wild type. Finally, the G292N/Y296V double variant showed a 5.3-fold increase in 3,5-diCP removal compared to wild-type *Tt*PPO (22.1% ± 0.9% and 4.2% ± 0.5%, respectively) (Fig. S4B). The same reactions were also performed using a 2 mM initial CP concentration, and the bioconversion yields increased 1.3 to 1.8 times in all cases.

## DISCUSSION

According to the JGI Genome Portal, T. thermophila harbors nine tyrosinase-like genes, six of which are expressed in the extracellular space. Transcriptome analysis revealed that out of these six genes, the one expressing protein ID 60685 is transcribed in higher levels when the fungus is grown on glucose, while it is the only one that has been identified in the secretome by mass spectrometry (52). It was also shown that this tyrosinase-like protein is induced by lignocellulosic substrates, like alfalfa straw.

The hypothetical protein was selected as a candidate tyrosinase, and the corresponding gene (*Tt60685*) was cloned and expressed in P. pastoris X-33. The recombinant enzyme was purified through IMAC, yielding approximately 40 mg of protein · liter^−1^ culture, which is 1.2 to 2 times higher than other fungal tyrosinases expressed previously in P. pastoris (26), A. niger (53), and E. coli (15, 17, 25).

Plant PPOs from various sources have been widely studied and reviewed (54), along with their structures (55); however, these enzymes are not discussed here, since they do not share many characteristics with the PPO described in this study.

Most fungal tyrosinase genes encode a protyrosinase protein, which is not active unless it is proteolytically cleaved at the C terminus. The proenzyme has a molecular weight of 66 to 71 kDa, while the active mature enzyme is 42 to 45 kDa, as reported in the literature (14, 17, 24, 25, 53, 56, 57). This maturation process occurs naturally in homologous expression (56, 57) or heterologous expression in eukaryotic hosts (26, 53), in contrast to prokaryotic hosts. The proenzyme produced in E. coli must be cleaved *in vitro* with trypsin to acquire its active form (17, 25), as it is not active when expressed from a gene encoding solely the mature form (14, 17). It is therefore assumed that the C-terminal amino acid sequence is essential for the correct folding of the enzyme. The only non-C-terminally processed fungal tyrosinase characterized so far is the catechol oxidase from A. oryzae (*Ao*CO), which shares the highest identity (45%) with *Tt*PPO. These enzymes undergo a proteolytic cleavage of several amino acids (51 amino acids [aa] for *Ao*CO or 64 aa for *Tt*PPO) at the N-terminal end by Kex2/furin-type proteases, which also cleave the signal peptide (49). It is unclear if that phenomenon takes place only in the expression hosts (P. pastoris for *Tt*PPO and T. reesei for *Ao*CO) or in the respective original hosts as well. For *Ao*CO, the theoretical molecular weight was 44.3 kDa, but two other bands appeared at 39.3 and 40.5 kDa on the SDS gel, which were also active, exhibiting specific activity similar to that of the full-length enzyme.

The optimum temperature (T_opt_) for some characterized fungal tyrosinases is 50°C (17, 19, 20). To our knowledge, *Tt*PPO exhibits the highest T_opt_ reported in literature. Furthermore, pH optima for most characterized tyrosinases typically range in the mildly acidic to neutral region (17, 19, 20, 26, 49), except for the T. reesei tyrosinase (pH 9) (56) and *Tt*PPO (pH 7.5 to 8). Additionally, *Tt*PPO exhibited superior thermostability compared to the fungal tyrosinase from T. reesei (56) and the bacterial ones from Rhizobium etli (20) and Ralstonia solanacearum (21) but similar thermostability to the A. oryzae catechol oxidase (half-life of 2 h at 60°C).

*Tt*PPO was able to oxidize all tested compounds at a certain extent, except l-tyrosine and *p*-hydroxyphenylacetic acid, showing great promiscuity to the substitution of the benzene ring and the size of the substrate. One conclusion that can be deduced from the substrate specificity of *Tt*PPO is that it prefers shorter side chains (e.g., vanillin > vanillic acid > ferulic acid) and/or that its activity is inhibited by the presence of a carboxyl group (e.g., pyrogallol > gallic acid). Furthermore, in the case of cresols, *Tt*PPO preferred the *ortho*-isomer over the *para*-isomer (2-fold more active). Interestingly enough, the recombinant PPO exhibited low activity on two typical peroxidase substrates, syringol and veratryl alcohol, which have not been studied previously with tyrosinases.

Recently reported PPO structures and mutagenesis studies have improved our understanding of this class of enzymes (1, 58). Catechol oxidase proved to be an inappropriate term for PPOs that are not able to oxidize tyrosine (2), as they do not just accept catecholic substrates. In our case, even though *Tt*PPO could not oxidize l-tyrosine, it had the ability to hydroxylate other monophenolic substrates, such as phenol, *ortho*-cresol, and tyrosol. Furthermore, it oxidized 1,8-dihydroxynaphthalene, which is commonly a precursor for the formation of melanins in the wild-type parental microorganism. Therefore, in this study, we confirmed that the hydroxylase activity is a general function of PPOs and should not be correlated with activity on tyrosine. The so-called catechol oxidase from A. oryzae also had a wide substrate range, including polyphenols (catechins), catecholic (catechol, 4-*tert*-butylcatechol, and caffeic acid), and phenolic (aminophenol, guaiacol, phenol, tyrosol, and *p*-cresol) derivatives and nonphenolics (aniline), even though it could not oxidize tyrosine and l-DOPA (49). Tyrosinase from T. reesei preferred *para*-substituted monophenols compared to their *ortho* isomers, and it could also oxidize aniline, which bears no hydroxyl groups on its ring (56). Last, tyrosinase from B. megaterium had a wide substrate range but showed lower activity on phenol derivatives (except tyrosine) than on the catechol derivatives (19). In most cases, the monophenolase-to-diphenolase ratio of such enzymes is calculated based on their activity on l-tyrosine/l-DOPA and is usually <1 (16, 17, 19, 57). The ratio of T. reesei tyrosinase calculated on phenol/catechol was 0.1 (56), 5 times higher than that for *Tt*PPO. An exception to these cases is a unique bacterial tyrosinase for which the monophenolase-to-diphenolase ratio is over 1 (21). Successful efforts to increase this ratio have been performed using mutagenesis techniques. Single mutants of tyrosinase from B. megaterium increased the ratio by 1.7 to 9 times (51, 59). However, our study focused on the effect of mutations on the increase of activity against CPs in order to generate a potent biocatalyst for bioremediation purposes.

PPOs, including tyrosinases and laccases, show great potential in environmental applications for the detoxification of wastewaters from phenolic compounds, since they have a wide substrate range and they require readily available O_2_ without any auxiliary cofactors. Laccases in particular have been extensively studied not just for their synthetic or industrial applications but also in the environmental field, especially for the decolorization of synthetic dyes (60, 61). On the other hand, very few reports on the potential of tyrosinases in the bioremediation field exist, which have been recently reviewed (33). Most studies focus on the immobilization of a commercially available Agaricus bisporus tyrosinase for the bioconversion of phenol, bisphenols, and *p*-cresol. Very few groups report the use of bacterial (from Bacillus and Streptomyces species) tyrosinases in their free (62, 63) or immobilized (64) form for the biotransformation of phenol and CPs. Streptomyces antibioticus tyrosinase was active on 3-CP and 4-CP but not 2-CP, as it proved to be a competitive inhibitor (63), in contrast to *Tt*PPO, which oxidizes 2-CP as well. The oxidation product for both CPs was identified as 4-chloro-1,2-*ortho*-quinone, which undergoes a nucleophilic aromatic substitution at the chlorine atom and forms a dimeric phenol-quinone adduct in the presence of excess phenol. Docking studies performed in the same study showed that coordination of both 3-CP and 4-CP occurs at CuB, and *ortho*-hydroxylation can only occur at the C-6 position. To our knowledge, the only reported production of PPO mutants with improved biotransformation capacity against CPs (4-CP in specific) is the recent work by Davis et al. (65). The authors produced a variant of a tyrosinase from Ralstonia solanacearum with an increased capacity to transform 4-halophenols to 4-halocatechols compared to the wild type (WT). The aim of their work was, however, the production of *o*-diphenols, which can be used as precursors for the synthesis of various chemicals.

The removal of phenolic pollutants using PPOs is based on the oxidation of phenols to the corresponding catechols, which are further oxidized to reactive quinones, which undergo spontaneous polymerization and precipitation. The purpose of the present work was to engineer *Tt*PPO variants with increased biotransformation capacity against various CPs. The rationale behind the choice of mutation sites was based on previous studies revealing amino acids that affect PPO specificity (12, 48, 66). Since there is no structure of a *Tt*PPO-CP complex available, the structural characteristics that determine the degrading capacity of *Tt*PPO mutants cannot be described in detail. As shown in Fig. 5, the L306A mutant has a more accessible CuA site than does the wild type. However, it shows similar or lower degrading capacity for all CPs. It could thus be assumed that Leu306 does not impede substrate binding, in spite of being a bulkier amino acid. In addition to that, a significant increase is observed in the oxidative activities of all variants that bear the G292N mutation against 3,5-diCP compared to the wild type. The asparagine residue found in this position in many PPOs has been shown to be required for monophenolase activity by coordinating a conserved water molecule that assists the deprotonation of monophenolic substrates (58). This suggestion was recently questioned by Kampatsikas et al. (67), who showed that two PPOs possessed tyrosinase activity despite lacking the asparagine residue. Our results confirm the implication of this residue in the substrate preference of PPOs, since all variants that bear the G292N mutation exhibit substantially altered activity against all CPs compared to the wild type.

*Tt*PPO and its variants were able to convert CPs at high concentrations (5 mM) that are usually toxic for microorganisms (34). The biotransformation yields for the tested CPs ranged from 21% to 35.4%, as measured by HPLC, and led to the formation of a brown precipitate. Even though the G292N/L306A variant seemed to have higher activity on 3-CP than the wild type based on the colorimetric assay, HPLC analysis showed that they consumed similar amounts of the pollutant. Nonetheless, residual chlorocatechol was detected only in the wild-type reaction, which could mean a lower bioremediation yield due to decreased precipitation. The highest increase in activity of a mutant compared to the wild type was achieved for the G292N/Y296V mutant on 3,5-diCP.

### Conclusions.

The present work describes the expression and characterization of a PPO from the thermophilic ascomycete fungus T. thermophila. This extracellular enzyme shares the highest homology (45%) with the catechol oxidase from A. oryzae. Its biochemical characterization showed that it is a thermostable enzyme with a broad substrate range, including CPs. In order to increase its activity on such substrates, single and double mutants were constructed. The mutagenesis sites were chosen based on previous studies that revealed amino acids affecting PPO specificity. In this study, we achieved the construction of a PPO double mutant (G292N/Y296V variant) with 5.3-fold increased activity on 3,5-diCP.

## MATERIALS AND METHODS

### Enzymes and chemicals.

KOD Hot Start DNA polymerase was purchased from Novagen (USA), while all other restriction enzymes were from TaKaRa Bio, Inc. (Japan). NucleoSpin gel, PCR cleanup, and NucleoSpin plasmid kits were supplied by Macherey-Nagel (Germany). Phenolic compounds used as the substrates were purchased from Sigma-Aldrich (USA).

### Strains, vectors, and media.

One Shot TOP10 Escherichia coli cells and Zero Blunt PCR cloning kit from Invitrogen (USA) were used for cloning work. P. pastoris (aka Komagataella phaffii) strain X-33 and pPICZαA vector were used for expression of the recombinant gene (Invitrogen).

E. coli cells were grown at 37°C in Luria-Bertani (LB) medium containing 50 μg kanamycin · ml^−1^ or 25 μg zeocin · ml^−1^ for selection of clones transformed with the pCR-Blunt or pPICZαA vector, respectively. P. pastoris was routinely cultivated at 30°C in either glycerol (BMG/BMGY) or 0.5% (vol/vol) methanol-containing (BMM/BMMY) buffered media, according to the instruction manual of EasySelect Pichia expression kit (Invitrogen).

Selection of P. pastoris transformants took place on YPDS plates containing sorbitol and zeocin at final concentrations of 1 M and 100 μg · ml^−1^, respectively. The WT strain of T. thermophila ATCC 42464 was maintained on 1.5% malt-peptone-agar slants at 4°C, and its total genomic DNA was isolated using the GenElute plant genomic DNA Miniprep kit from Sigma-Aldrich (USA).

### DNA manipulation techniques and transformation of P. pastoris.

The gene coding for the hypothetical protein *Tt*PPO (protein ID 60685; chromosome_6, 1676944 to 1678961, excluding the native signal peptide) was amplified from the genomic DNA by PCR, using primers 60685-F/60685-R (Table 4), designed according to the available sequence (DOE Joint Genome Institute; http://genome.jgi.doe.gov/), which included the EcoRI and XbaI restriction sites at the respective 5′ ends. DNA amplification was performed by a KOD Hot Start polymerase for 35 cycles of denaturation (94°C for 15 s), annealing (61°C for 30 s), and extension (68°C for 100 s), prior to an initial denaturation at 95 °C for 2 min and followed by a further extension for 2 min at 68°C. PCR products were directly cloned into pCR-Blunt vector according to the Zero Blunt PCR cloning kit, and their sequences were determined. Intron removal was performed by the overlap extension-PCR (OE-PCR) method, as described by Dimarogona et al. (68). Recombinant plasmid pCR-Blunt/*Tt60685* was used as the template at an appropriate dilution for the amplification of each exon by the KOD Hot Start polymerase. The PCR programs were as follows: for the first exon, primers 60685-F/60685e-R at 94°C for 2 min, followed by 35 cycles of 94°C for 15 s, 61°C for 30 s, and 68°C for 30 s, with a final extension step at 68°C for 2 min, while for the second exon, primers 60685e-F/60685-R were used at 94°C for 2 min, followed by 35 cycles of 94°C for 15 s, 61°C for 30 s, and 68°C for 70 s, with a final extension step at 68°C for 2 min. Subsequently, the two fragments were fused by a PCR using the external primers 60685-F/60685-R, under the conditions described for the first amplification, only the extension step lasted 80 s instead of 100 s. The final PCR cleaned-up product was sequenced after cloning into the pCR-Blunt vector.

**TABLE 4:** Primer sets used in this study for the amplification, *in vivo* splicing, and site-specific mutagenesis of the *Tt60685* gene from T. thermophila

Primer name	Nucleotide sequence (5′→3′)^*a*^	Length (nt)
60685-F	GC*GAATTC*CGCTGTTCTTCCGATGCGCC	28
60685-R	GC*TCTAGA*TAAAAGTAGCACAGCTCGCC	28
60685e-F	CGGGTCTCTGTCCAAGGATG	20
60685e-R	CTTGGACAGAGACCCGTATTCGCGGCGAAAGATGAGC	37
G292N-F	GAGATCGGCCCCCAC**AA**CGGCATCCACTACAC	32
G292N-R	GTGTAGTGGATGCCG**TT**GTGGGGGCCGATCTC	32
L306A-F	CGGGCGGCGAC**GCA**TTCACCTCCCCCGG	28
L306A-R	CCGGGGGAGGTGAA**TGC**GTCGCCGCCCG	28
Y296V-F	CGGCATCCAC**GTA**ACCATCGGCGGCGACCC	30
Y296V-R	GGGTCGCCGCCGATGGT**TAC**GTGGATGCCG	30

a The restriction sites introduced in the primer sequence are presented in italics (EcoRI, GAATTC, and XbaI, TCTAGA). Underlined sequence denotes the area of homology between each primer and its oppositely oriented overlapping partner. Nucleotides altered for mutagenesis are presented in bold.

The *Tt60685* gene was gel purified after digestion with restriction enzymes EcoRI and XbaI and then ligated with the doubly digested pPICZαA vector in-frame with the α-secretion factor and the C-terminal His_6_ tag. Cloning success was confirmed after sequencing the recombinant pPICZαA/*Tt60685* plasmid, which was amplified in E. coli TOP10F′ cells resistant to 25 μg zeocin · ml^−1^. The correctly recombinant plasmid was linearized with PmeI restriction enzyme and transformed into P. pastoris X-33 cells by electroporation, as described in the EasySelect Pichia expression kit instruction manual.

### Screening of recombinant P. pastoris transformants, expression study, and purification of recombinant T*t*PPO.

Thirty-two transformed colonies grown on YPDS plates (100 μg zeocin · ml^−1^) were plated out on a methanol medium (MM) plate (1.34% [wt/vol] yeast nitrogen base, 4 × 10^−5^% [wt/vol] biotin, and 0.5% [vol/vol] methanol) and incubated at 30°C for 3 days. Subsequently, 3 ml of 10 mM 4-chlorocatechol prepared in 100 mM sodium phosphate (pH 7) buffer was poured gently and uniformly on the plate, which was then incubated at 60°C for 15 to 30 min. The oxidized dark-brown products of 4-chlorocatechol appeared around and on the yeast colonies and were indicative of the amount of enzyme produced by each recombinant strain. Based on color formation, seven colonies were picked for further expression studies in liquid cultures. BMG medium was inoculated with a colony and incubated at 30°C and 200 rpm for 20 h. Grown cells were used to inoculate BMM medium at a final optical density at 600 nm (OD_600_) of 1 and incubated for 4 days under the same conditions. Each day, the extracellular medium was assayed for PPO activity compared to the WT X-33 strains. The transformant which exhibited the highest PPO activity was used for further studies.

The effect of the added CuSO_4_ (0 to 100 μM) on the PPO activity in buffered minimal medium containing glycerol or methanol (BMG/BMM) was studied in correlation with the cell growth after 4 days of induction. Furthermore, the effect of the type of induction medium (mineral-BMM versus complex-BMMY) in correlation with the induction temperature (23 versus 30°C) was also studied.

Recombinant *Tt*PPO was isolated from 2 liters of culture broth after 4 days of induction at 23°C and 200 rpm. The broth was concentrated, and the recombinant protein was isolated using an immobilized metal ion affinity chromatography (IMAC) column, as described previously (69).

Sodium dodecyl sulfate-polyacrylamide gel electrophoresis (SDS-PAGE) was performed to examine the homogeneity and molecular weight of the purified enzyme. In order to investigate potential *N*-glycosylation on the recombinant protein, endoglycosidase treatment was performed by Endo H enzyme (NEB, USA) under native conditions, according to the manufacturer's manual.

Activity staining on SDS-PAGE gels was performed for samples that had not been denatured. After running the samples, the gel was washed twice with distilled water and then immersed in 2.5 mM 4-chlorocatechol prepared in sodium phosphate buffer (pH 7). The gel was incubated for approximately 30 min at ambient temperature until dark-brown bands appeared.

### Characterization of recombinant *Tt*PPO.

A typical enzymatic assay was performed in a SpectraMax 250 microplate reader (Molecular Devices, USA) set at 40°C. The final reaction volume was 250 μl, containing 230 μl of 5 mM 4-chlorocatechol in 0.1 M sodium phosphate buffer (pH 7) and 20 μl of enzyme. An increase in absorbance at 440 nm (*A*_440_) was recorded for 20 min. One unit (U) of enzymatic activity was determined as 1 Δ*A*_440_ · min^−1^.

Optimum temperature was determined under the standard assay conditions at various temperatures from 30 to 70°C, while the thermostability of the recombinant enzyme was determined by assaying the residual activity after incubation at 40 to 70°C for 0.5 to 24 h. Optimum pH was determined as relative activity by assaying the activity in the pH range of 3 to 9 in different buffer systems, as follows: 0.1 M phosphate-citrate (pH 3 to 6), 0.1 M sodium phosphate (pH 6 to 8), and 0.1 M Tris-HCl (pH 8 to 9).

The substrate range of the purified enzyme was determined by incubating 15 μg *Tt*PPO in 1 ml of 2 mM substrate (in 0.1 M sodium phosphate buffer [pH 7]) for 20 h at 35°C. The UV/Vis spectrum of the reactions was recorded and compared to the respective controls. The activity for each substrate was calculated as the Δ*A* at the wavelength where the products show their maximum absorbance (λ_max_). For most products, the λ_max_ was 400 nm, except for the following: 4-CP and catechin, 440 nm; resorquinol, 450 nm; syringol, 470 nm; l-DOPA 475 nm; epicatechin, hydroquinone, and 3,5-diCP, 480 nm; and phenol, 530 nm. The substrates for which the enzyme had high activity were tested under the standard assay conditions to calculate either the specific activity of the enzyme or its kinetic parameters. For each substrate, 1 unit (U) was calculated as 1 Δ*A*_λmax_ · min^−1^.

Kinetic studies of the purified enzyme were performed by assaying various concentrations of 4-chlorocatechol (0 to 5 mM), catechol (0 to 60 mM), catechin (0 to 10 mM), l-DOPA (0 to 7 mM), vanillin (0 to 5 mM), guaiacol (0 to 15 mM), and hydroquinone (0 to 70 mM). Kinetic constants were estimated using a nonlinear regression model in Prism 5 from GraphPad Software, Inc. (USA).

Protein amount of purified enzyme was quantified through *A*_280_ measurements (70) using a molar extinction coefficient of 75,540 M^−1^ · cm^−1^, calculated with the ProtParam tool from ExPASy (71).

### Site-directed mutagenesis.

The *Tt*PPO mutants were prepared by following the instructions of QuikChange site-directed mutagenesis kit (Stratagene) using pPICZαA/*Tt60685* vector as the template and primer pairs shown in Table S1. The correct sequence of the mutated gene was confirmed by DNA sequencing, after which the plasmids were transformed by electroporation to P. pastoris, as described above.

### Protein sequence alignment and homology modeling.

Multiple-sequence alignment was performed using the MEGA7 software (72) with the ClustalW option. A phylogenetic tree was constructed using the maximum likelihood method in the same software. Prediction of the signal peptide was done on the SignalP 4.0 server (73). Prediction of glycosylation sites was performed using the NetNGlyc 1.0 (74) and NetOGlyc 4.0 (75) servers. The theoretical molecular weight and isoelectric point of the recombinant protein were estimated by ProtParam.

For the identification of sequence homologues with known structures and the construction of a structural model, the *Tt*PPO sequence was submitted to the HHpred server (toolkit.tuebingen.de/hhpred). The search was performed against the PDB database using default settings. Using the top-scoring PDB hit, a homology model was built using the Modeler software (76) through the HHpred interface. The stereochemical quality of the model was evaluated through Ramachandran plots produced using the program RAMPAGE (77).

### Bioconversion of CPs.

Phenol, mono-CPs, and di-CPs were used as the substrates at a final concentration of 5 mM in reactions with the wild-type *Tt*PPO and its mutants. The reactions took place in an Eppendorf Thermomixer comfort at 30°C/900 rpm for 16 h. After that period, the absorption of the reaction mixture was measured with respect to their control reactions. The results were normalized for each mutant, taking into consideration the concentration of the enzyme used in each reaction.

For each substrate, the best bioconversion candidate was selected and compared to the wild-type PPO for the ability to consume the corresponding CP. Reactions (1 ml) with 2 and 5 mM initial substrate concentrations were analyzed 20 h after the addition of 0.09 mg of the corresponding *Tt*PPO variant. After that time, enzyme activity was terminated by the addition of 1 N HCl (0.1 ml), and samples were analyzed after centrifugation for the removal of the formed precipitate. Quantification of CPs was done using an HPLC method based on the one described by Davis et al. (65). The apparatus used in our case was a Shimadzu LC-20AD unit equipped with a SIL-20A autosampler. A C_18_ reverse-phase Nucleosil 100-5 column (Macherey-Nagel, Germany) was used at a flow rate of 0.8 ml · min^−1^. The analysis of mono-CPs lasted 15 min and for di-CPs lasted 30 min.

## Supplementary Material

Supplemental file 1
