# Supplementary material for: Versatile Fungal Polyphenol Oxidase with Chlorophenol Bioremediation Potential: Characterization and Protein Engineering
Source: Appl Environ Microbiol. 2018 Nov 15;84(23):e01628-18. doi: 10.1128/AEM.01628-18 (PMC6238066; doi:10.1128/AEM.01628-18)
Supplement: Supplemental file 1 [file zam023188873s1.pdf]

Electronic Supplementary Material

Title: **Characterization and protein engineering of a novel versatile fungal polyphenol oxidase with chlorophenol bioremediation potential**

Authors: **Efstratios Nikolaivits[1], Maria Dimarogona[2], Ioanna Karagiannaki[1], Angelina Chalima[1], Ayelet Fishman[3] and Evangelos Topakas[1,4*]**

Affiliations:

[1]Biotechnology Laboratory, School of Chemical Engineering, National Technical University of Athens, 15780 Athens, Greece

[2]Department of Chemical Engineering, University of Patras, 26504 Patras, Greece

[3]Department of Biotechnology and Food Engineering, Technion-Israel Institute of Technology, Haifa, 3200003, Israel

[4]Biochemical and Chemical Process Engineering, Division of Sustainable Process Engineering, Department of Civil, Environmental and Natural Resources Engineering, Luleå University of Technology, SE-97187 Luleå, Sweden

***Corresponding author's e-mail:** vtopakas@chemeng.ntua.gr

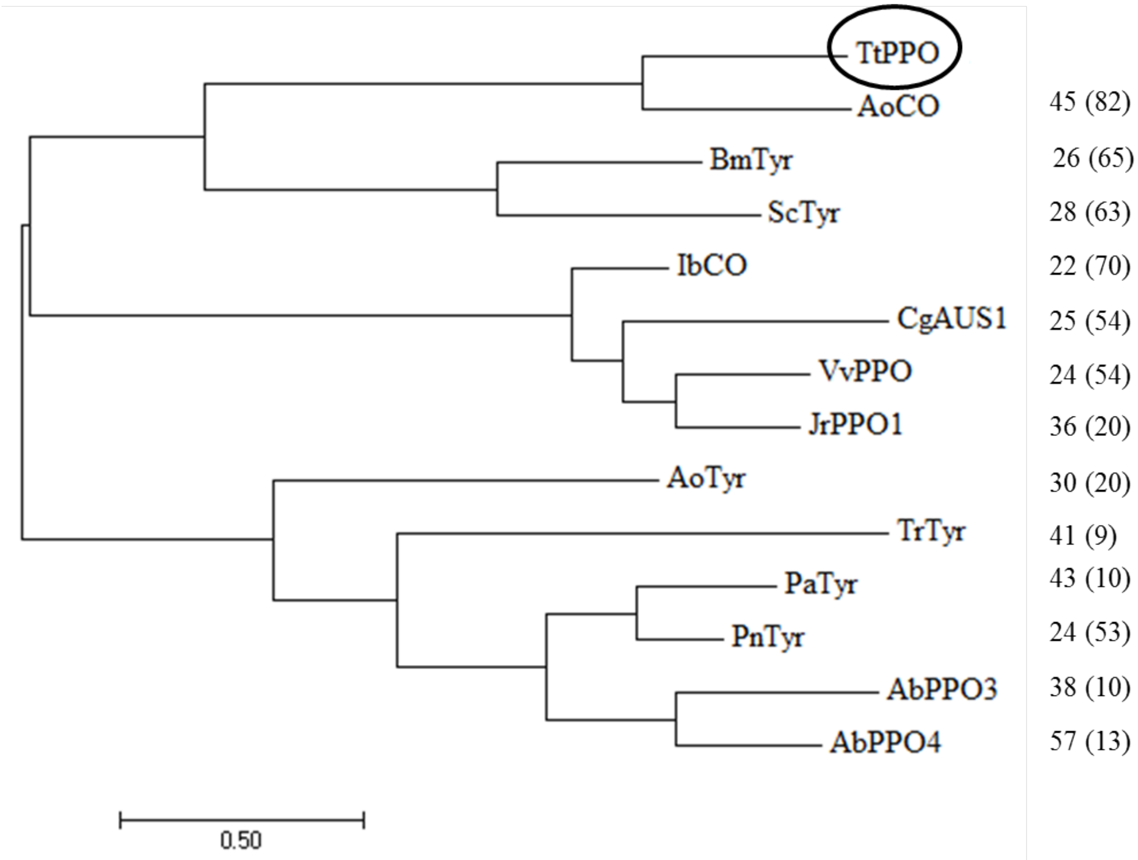

Fig. S1 Phylogenetic tree based on known PPO sequences from different classes of organisms. Identity and query cover (in brackets) are presented as percentages (%) for each sequence according to the results of the BLAST compared to *Tt*PPO. Abbreviations: PPO, polyphenol oxidase; CO, catechol oxidase; Tyr, tyrosinase; AUS, aurone synthase; Ao, *Aspergillus oryzae*; Bm, *Bacillus megaterium*; Sc, *Streptomyces castaneoglobisporus*; Ib, *Iponomea batatas*; Cg, *Coreopsis grandiflora*; Vv, *Vitis vinifera*; Jr, *Juglans regia*; Tr, *Trichoderma reesei*; Pa, *Polyporus arcularius*; Pn, *Pholiota nameko*; Ab, *Agaricus bisporus*.

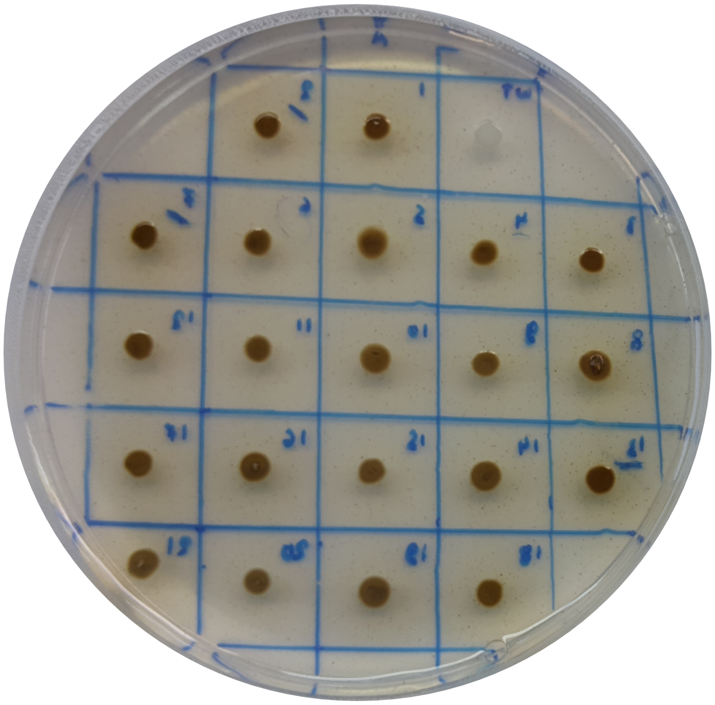

Fig S2. Plate assay of Zeocin™ resistant transformants compared to the wild type *P. pastoris* X-33 strain (top right square) for detection of PPO activity.

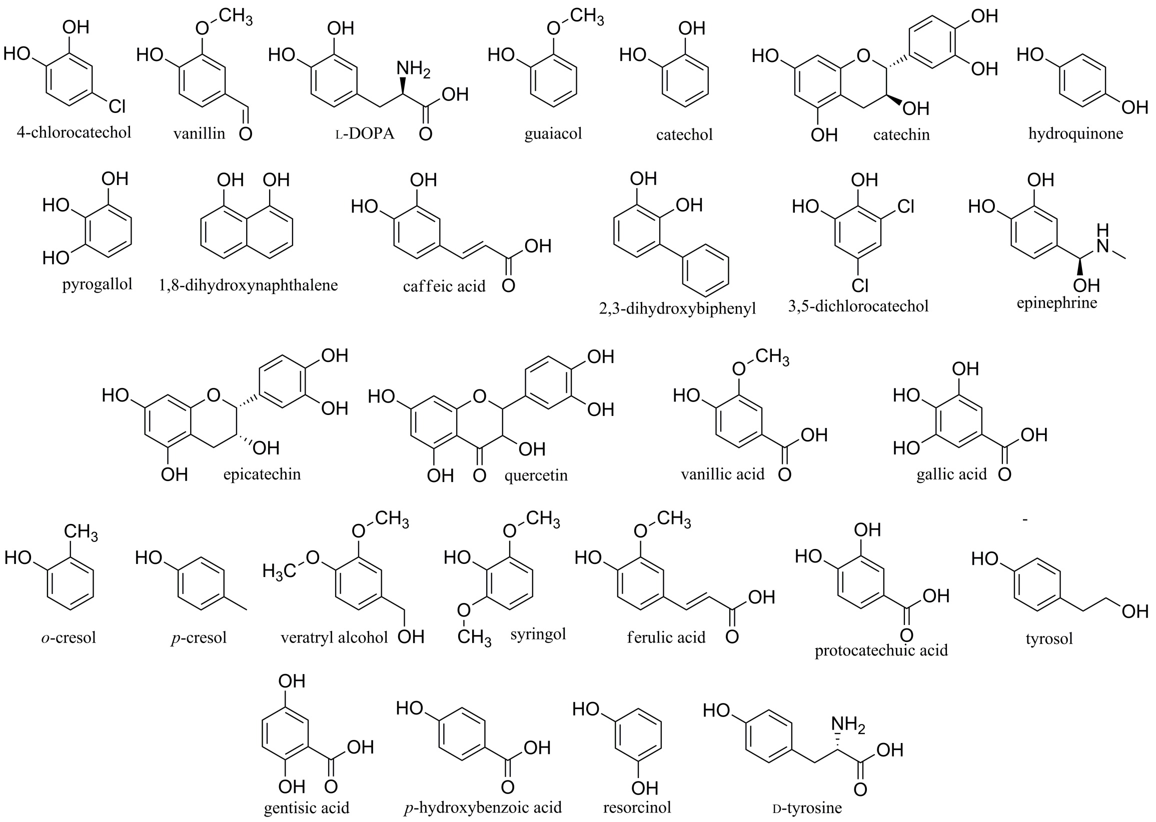

Fig. S3 Chemical structures of the compounds *Tt*PPO accepts as substrates, roughly placed according to the activity it presents on each of them.

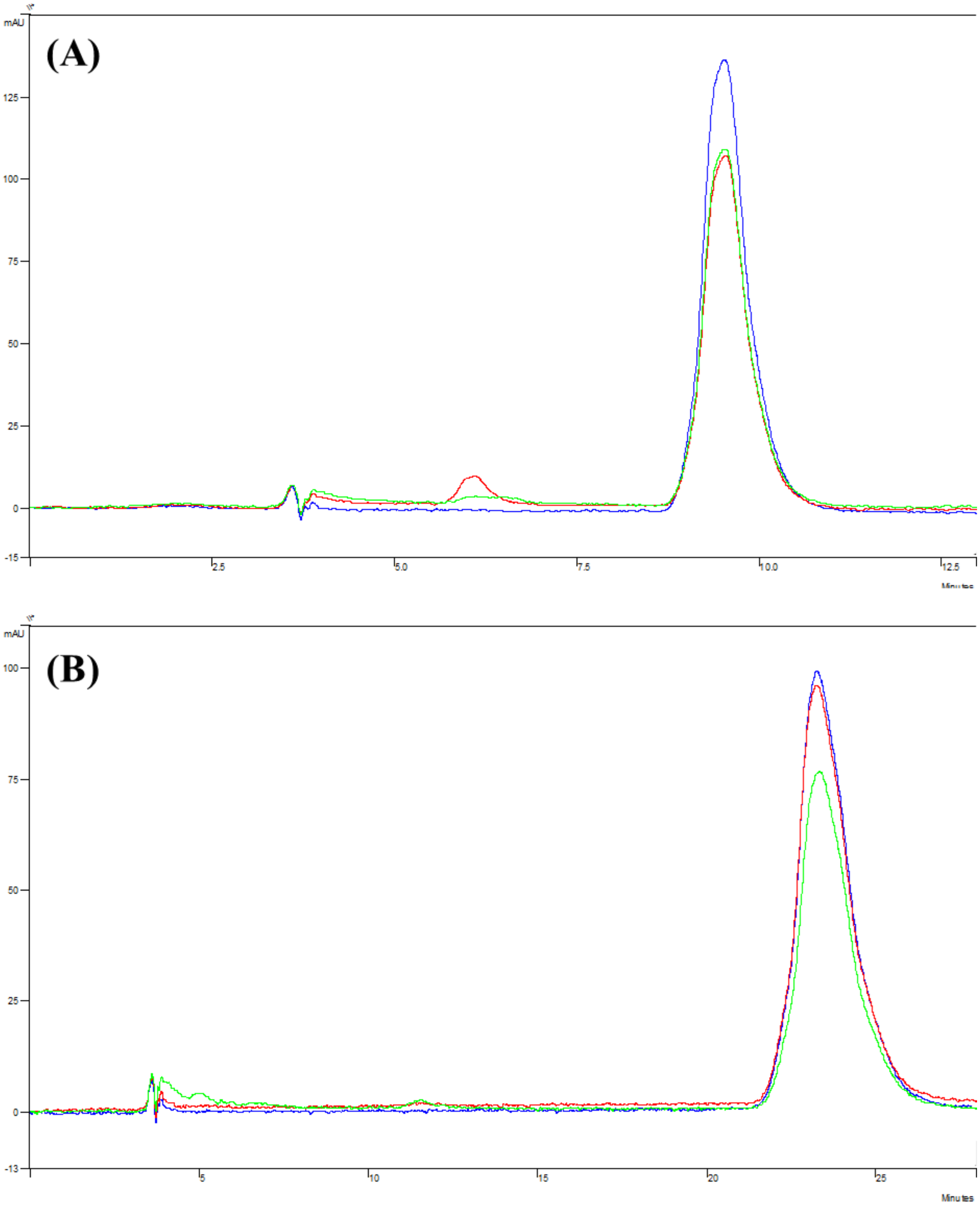

Fig. S4 HPLC chromatograms of (A) 3-CP reaction (blue: control, red: wild-type *Tt*PPO, green: variant G292N/L306A) and (B) 3,5-diCP reaction (blue: control, red: wild-type *Tt*PPO, green: variant G292N/Y296V)
